# Biophysical Properties of Foamed and Solid Polymers Used in Orthotics and Prosthetics

**DOI:** 10.3390/ma14226877

**Published:** 2021-11-15

**Authors:** Klemen Bohinc, Anže Abram, Anamarija Zore, Roman Štukelj, Ana Lenarčič, Rajko Vidrih, Andrijana Sever Škapin

**Affiliations:** 1 Faculty of Health Sciences, University of Ljubljana, Zdravstvena pot 5, 1000 Ljubljana, Slovenia; zorea@zf.uni-lj.si (A.Z.); stukeljr@zf.uni-lj.si (R.Š.); ana.lenarcic99@gmail.com (A.L.); 2 Department for Nanostructured Materials, Jožef Stefan Institute, Jamova cesta 39, 1000 Ljubljana, Slovenia; anze.abram@ijs.si; 3 Biotechnical Faculty, University of Ljubljana, Jamnikarjeva 101, 1000 Ljubljana, Slovenia; Rajko.Vidrih@bf.uni-lj.si; 4 Slovenian National Building and Civil Engineering Institute, Dimičeva ulica 12, 1000 Ljubljana, Slovenia; andrijana.skapin@zag.si; 5 Faculty of Polymer Technology-FTPO, Ozare 19, 2380 Slovenj Gradec, Slovenia

**Keywords:** surface characterization, bacterial adhesion, prosthetic and orthotic material, surfaces, foamed materials

## Abstract

Orthotic and prosthetic materials should have good mechanical and antibacterial properties. Therefore, in our study, we consider four common foamed closed-cells and two solid polymeric materials regarding their mechanical behaviour and tendency for bacterial adhesion. For all materials, the surface roughness, hydrophobicity, zeta potential, tensile properties, hardness and CIE color parameters were measured. We found that foamed polymeric materials have higher roughness, higher hydrophobicity, lower Young’s modulus, lower maximum tensile strength and lower hardness than solid materials. Bacterial adhesion test measurements based on observation by scanning electron microscopy show much a lower adhesion extent of *S. aureus* on solid materials than on foamed materials. The measured biophysical properties could be the key data for users to select the optimal materials.

## 1. Introduction

Due to high demand for light and durable materials in orthotics and prosthetics in the last 60 years there has been great technological progress. Many new materials with mechanical properties suitable for orthotics and prosthetics use have also been developed. The new polymer materials thus led to revolutionary advances that enabled greater durability and strength as well as more cosmetic improvements. Polypropylene (PP) and polyethylene (PE) are semi-crystalline polymers and the most often polymers used in many fields of industry. They are known as polymers with desired properties, for example low density, recyclability, quite high thermal stability, and good chemical resistivity [1]. Many studies have been focused on the effect of the morphological properties of certain polymers on their mechanical properties [2]. By the addition of fillers or reinforcements mechanical properties of polymers can be significantly improved [3]. Kennedy et al. [4] showed how the tensile properties of the linear PE depend on its structural factors, while Amjadi et al. [5] studied the effect of processing technique and measuring conditions on tensile behavior of high-density PE (HDPE). They found that the processing technique and thickness of samples significantly affect the tensile properties of HDPE.

Foaming of polymers enables reduction of weight, what provides new properties of material and is welcome from the economic point of view as well [6]. Recent developments allow producing foams with defined pores that consequently expand the possibilities for their applications. Many researchers reported on the tensile properties of different foamed polymers [7,8,9]. There was found a strong dependence of microscopic foam structures, density and molecular weight on its mechanical properties [7,10,11]. The foam toughness can be also increased by increasing its density and molecular weight. It was reported that by tailoring the microstructure of foams desired comprehensive materials properties could be achieved [12].

Orthoses and prostheses are mobility assistive utilities often in touch with patient’s skin. Inner sides of orthotic devices are in direct contact with patient’s skin [13], which acts as a physical barrier to the patient’s body against the environment like microorganisms or toxic substances. Among microorganisms, bacteria, fungi and viruses can colonize to the orthotic surface. Some microorganisms are harmless and, in some cases, provide vital functions of the skin [14]. The skin’s permanent microbiota includes staphylococci, most notably *Staphylococcus (S.) aureus* and *S. epidermidis* [15]. They are commensal on the human skin and some strains have a possibility of surface adhesion und subsequent biofilm formation. Biofilms can cause infections if coming in contact with the impaired skin barrier [16].

Bacterial adhesion to the material surface takes place when the bacteria are strongly attached to the surface [17]. The phenomenon of bacterial adhesion to the surface of materials is classified as non-specific and specific. The first phase involves non-specific adhesion, which is the result of the physicochemical interactions between the bacterial cell wall and the material’s surface. These interactions include electrostatic forces, Van der Waals forces, and hydrophobic forces and represent the first step of the adhesion, where the adhesion is still reversible [18]. The second adhesion phase is driven by specific forces between surface structures of bacteria and material surfaces like bacterial surface polymer structures including capsules, fimbria (or pili) and mucus which consist of polysaccharides and proteins that act as bacterial adhesins [19].

Biofilm formation can cause different types of infection. The inhibition of bacterial adhesion can be obtained by appropriate material surface characteristics. Crucial for the bacterial adhesion are surface characteristics like surface energy, roughness, wettability, and zeta potential [17]. These characteristics can be measured by techniques like profilometry, atomic force microscopy, tensiometry, and electrophoresis. Changing surface characteristics make it possible to gain new knowledge about the bacterial adhesion and subsequent reduction of microbial colonization on different type of material surfaces can be reached [18].

Orthoses are thoughtfully designed often with patient collaboration to enable the patient comfort needed for every activity [20]. Regarding patient comfort, orthoses are designed to relieve pain, protect vulnerable tissues, and provide joint stabilization and emotional well-being [20]. Beside the aforementioned requirements orthoses must fulfil, their color should mimic skin color as closely as possible.

The color can be objectively quantified by spectrophotometers or standard organization system like Commission Internationale de l’Eclairage (CIE) [21]. The CIE LAB shows color’s lightness (L*), red/green intensity (a*) and yellow/blue intensity (b*). Positive L*, a*, b* parameters correspond to lighter, reddish and yellowish color respectively, while lower or negative values mean darker, greener or bluer color. The CIE L*, a*, b* color has been successfully applied for evaluating the color of different surfaces [21,22,23]. Beside basic L*, a* and b* parameters hue angle (also called color appearance parameter) ranges from 0 to 360°. It is obtained as arctan(b*/a*), 0° depict red, 120° green and 240° blue note. Chroma or relative saturation (C*) is obtained as (a*^2^ + b*^2^)^1/2^ and is the perceived strength of a surface color, the degree of visual difference from a neutral grey of the same lightness.

The purpose of this investigation was to examine mechanical and antibacterial surface characteristics of orthotic and prosthetic polymeric materials, like foamed plastic, i.e., PE foamed, EVA foamed (poly(ethylene-vinyl acetate)) and cork, as well as solid HDPE and PP. Mechanical tests, namely tensile and hardness tests were used to follow the mechanical properties of selected orthotic and prosthetic polymeric materials. The bacterial adhesion extent of *S. aureus* on all studied surfaces was also determined. For a better understanding of tendency for bacterial adhesion, we also measured the surface properties like roughness, hydrophobicity, and electric zeta potential. For all materials, we determined the CIE color parameters with the aim to determine how closely they match the human skin color.

## 2. Materials and Methods

### 2.1. Materials

#### 2.1.1. Substrate

Six different polymeric materials commonly used in prosthetics and orthotics were selected in this study (Table 1). Out of six, four materials were foamed closed-cells polymeric materials, whereas two polymeric materials were solid. The density of polymeric materials was determined according to ISO 1183-1:2019. For each sample, 3 measurements were performed (standard deviation of results was up to 1%). To measure the bacterial adhesion, surface roughness and water contact angle, each material was cut to the dimensions of 2 cm × 2 cm, thickness 7 mm, while for measuring the zeta potential the dimensions of samples were 1 cm × 2 cm. The coupons were purified with 70% ethanol, irradiated with UV light for 30 min each side was transferred into six-well microtiter dishes.

#### 2.1.2. Bacteria

*Staphylococcus (S.) aureus* is often part of the skin microbiota. It is a facultative anaerobe, Gram-positive cocci (round-shaped) and form grapes like structures. *S. aureus* can infect almost any tissue in the body. Frequent contaminate catheters and surgical implants, where it forms biofilms. In the study we used the standard isolate *S. aureus* ATCC 25923 (CCM = Czech Collection of Microorganisms 3953, Brno, Czech Republic).

Pure culture of bacteria was cultivated on blood-agar plates at 37 °C for 24 h under aerobic conditions. From blood-agar cultures we prepared overnight culture by inoculating one third of surface of microbiological loop of culture from blood-agar into BHI (Brain-Heard Infusion-Biolife Italiana S.r.l., Milan, Italy—(4012302)), edium and incubated it overnight for 18 h at 37 °C to reach concentration 10^9^ bacteria/mL. This suspension of overnight culture was diluted in the ratio 1:30 and co-incubated with our samples of prosthetic material for 14 h at 37 °C according to bacterial growth curves. Prosthetic material samples were sterilized with UV light before incubation. After incubation samples were rinsed three times with PBS (1X Phosphat Buffered Saline) and three times with water.

### 2.2. Methods

#### 2.2.1. Surface Morphology and Roughness

We used Form Talysurf Series 2 (Taylor-Hobson Ltd., Leicester, UK) profilometer to measure the topography for orthotic and prosthetic surfaces. The resolution was 0.25 μm, 1 μm and 3 nm in the *x*, *y*, and *z* directions, respectively. A set of parallel line scans was performed with a tip of 2 μm. Data were processed using TalyGold, Taylor Hobson, Leicester, UK. To exclude the waviness from roughness, we apply a Gaussian cut-off filter of 0.8 mm. The imaging data serve for the quantitative determination of surface characteristics from which we calculate the arithmetic average roughness (*R*_a_) and root mean square roughness (*R*_q_). For each type of orthotic and prosthetic surface, three-line measurements in the length of 5 mm were performed.

#### 2.2.2. Zeta Potential Measurements

We measured the zeta potential of the orthotic and prosthetic surfaces with the electro-kinetic analyser (SurPASS, Anton Paar GmbH, Graz, Austria). At standard conditions 1 mM phosphate-buffered saline (PBS) solution was forced to flow through a capillary and the electrical (streaming) potential between the ends of the capillary was produced. We calculated the zeta potential from the streaming potential by applying the Helmholtz–Smoluchowski equation.

#### 2.2.3. Contact Angle Measurements

We used an Attension Theta (Biolin Scientific, Gothenburg, Sweden) tensiometer to measure the contact angle between water droplet and the surface. Orthotic and prosthetic surfaces were put on the sample stage and a water droplet was placed on the material surface and the contact angle between the water droplet and the material surface was determined. To improve the statistics several measurements were performed. Finally, we calculated the surface free energy from the measured advancing and receding contact angles.

#### 2.2.4. Mechanical Properties: Tensile and Hardness Tests

The specimens for the tensile measurements were prepared according to EN ISO 527-2:2012, type 5×. Tensile properties of selected materials were measured according to EN ISO 527-3:2018 using Z100/SN5A apparatus (Zwick, Ulm, Germany) at 23 °C and a relative humidity of 50%, and strain rate of 2 mm/min. For each paddle-shaped sample, five measurements were taken. The initial distance between the grips and the initial gauge length was 20 mm and 10 mm, respectively. A preloading of 1 N was applied.

The hardness of the material was measured using a shore A durometer (Zwick, Ulm, Germany). The measured values indicate the resistance to indentation of the tested material on a scale between 0 and 100. For each sample, five measurements were taken.

#### 2.2.5. Monitoring of Bacterial Adhesion on Surfaces

The adhesion of *S. aureus* to surfaces was made by the procedure described by Bohinc et al. [23] and Gorjan et al. [24] with modifications. First, we immersed each specimen into the diluted (1:30) overnight culture of *S. aureus* with BHI broth. Plates were incubated for 10 h and afterwards the attached bacteria were fixed with 0.1 M PBS and hot air. At the end, specimens were washed in distilled water and hot air-dried.

Bacterial adhesion was detected by a Field emission scanning electron microscope (FESEM) (Zeiss ULTRA plus, Carl Zeiss, Germany). We used an InLens detector. The samples were placed on double-sided carbon tape. They were also Pt-coated (thickness 3 nm) prior to examination using Gatan, PECS, Model 682 (Precision Etching and Coating System, Gatan, Pleasanton, CA, USA).

#### 2.2.6. Color Measurements of Orthotic Materials

A reflectance spectrophotometer (CR-400; Minolta, Kyoto, Japan) was used for determining the color of the orthotic material surfaces using the CIE L*, a*, and b* parameters. L* ranges from light to dark, a* from red to green and b* from yellow to blue. The CIE L*, a* and b* parameters were carried out five times on each orthotic material. Besides basic parameters, the chroma (C*) was given as (a*2 + b*2)^1/2^ and the hue angle (°) was obtained as arctg(b*/a*).

## 3. Results

### 3.1. Surface Morphology/Topography and Roughness

**Figure 1:**
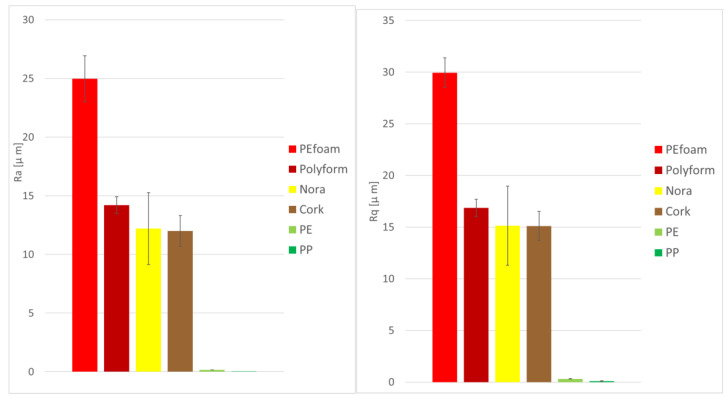
Roughness R_a_ and R_q_ of six material surfaces: PEfoam, Polyform, Nora, cork, PE and PP.

Figure 1 presents the surface roughness of six materials measured by mechanical profilometer. Solid material surfaces show very low surface roughness whereas foamed materials have roughness one order of magnitude larger than the solid materials. The arithmetic average roughness R_a_ of polyethylene surface is (161 ± 7) nm whereas the polypropylene surface has roughness (48 ± 8) nm.

**Figure 2:**
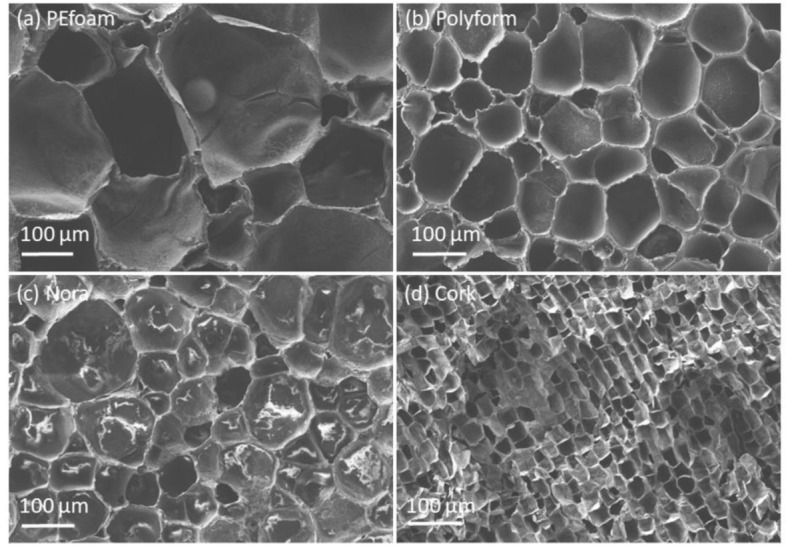
Field-emission scanning electron microscopy (FE-SEM) micrographs of cellular structure of foamed samples: (**a**) PEfoam, (**b**) Polyform, (**c**) Nora and (**d**) cork.

Figure 2 shows FE-SEM micrographs of cellular structure of foamed samples: PEfoam, Polyform, Nora and cork. The corresponding estimated cell sizes are 200 μm, 100 μm, 50 μm and 20 μm.

### 3.2. Zeta Potential

**Figure 3:**
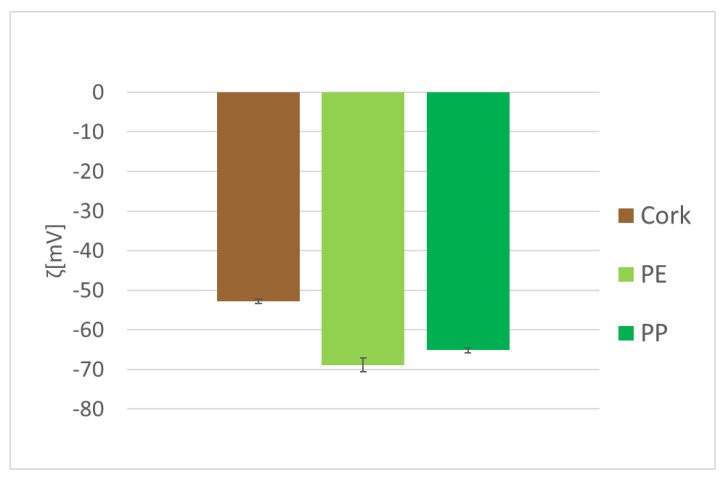
Zeta potentials of three different material surfaces: cork, PE and PP.

The measured zeta potential shows that material surfaces were negatively charged. The results for three materials are shown in Figure 3. The zeta potential was within the range between −53 mV and −69 mV. The zeta potential measurements on the foamed materials were not taken due to physical limitations of the experimental setup (problems with adhesion and sealing of the measuring cell).

### 3.3. Contact Angle Measurements

**Figure 4:**
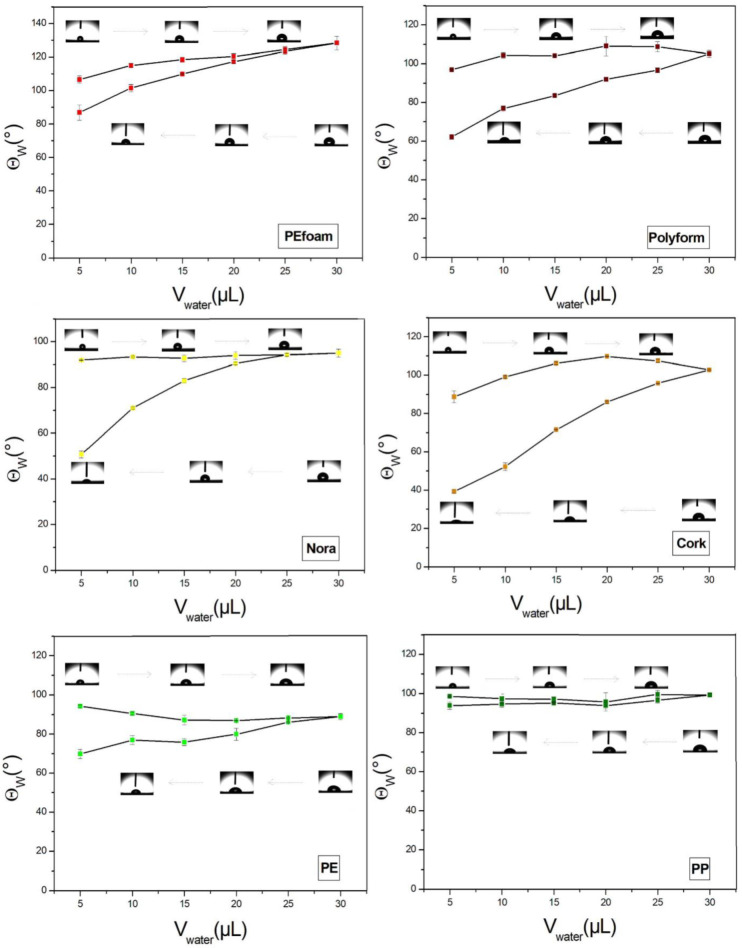
Contact angles of water droplet on six different materials: PEfoam, Polyform, Nora, cork, PE and PP. Advancing (upper curves) and receding (lower curves) contact angles are shown. Insets show liquid droplets on the material surfaces.

With an optical tensiometer, the contact angles of a water droplet on different orthopedic material surfaces were measured. For Θ > 90° surfaces are hydrophobic and for Θ < 90° surfaces are hydrophilic. For each material few several measurements were performed from which we calculated the average contact angle with its standard deviation. Figure 4 shows contact angles of six different orthopedic surfaces. The surfaces of closed-cell materials are hydrophobic. The highest surface free energy was in PE, whereas the lowest free energy was observed for PEfoam (Table 2).

### 3.4. Mechanical Properties

Figure S1 presents tensile curves for selected orthotic and prosthetic materials. The plots display the stress (the load divided by the initial cross-sectional area) as a function of the elongation. Typical stress/strain curves corresponding to all samples are shown in Figure S1a–f. The mean values of five parallel measurements of different selected tensile properties are presented in Table 3. In the case of solid polymers (PE and PP samples; (Figure S1e,f) the yield strength (σ_Y_) and the yield strain (ε_Y_) are close together for all parallels; they are 21 MPa and 10% for PE and 33 MPa and 4.0% for PP sample. But the maximum tensile stress (σ_M_) for the PE sample is achieved only near the break (28.3 MPa; 470%), while for PP sample σ_M_ is actually σ_Y_ (33 MPa). The strain at break (ε_B_) for the PP sample scatters among specimens from 23% to 89%. It can also be deduced from Table 3 and Figure S1a–d that all the remaining four samples have quite similar shapes of tensile measurement curves. The maximum tensile stress (σ_M_), the nominal strain value at maximum tensile stress (ε_M_) and the strain at break (ε_B_) were all near the end of the curves, near the break that occurred. The Young’s modulus of 2250 MPa is the largest for PP, following 1380 MPa for PE. Foamed polymeric materials have lower values of the Young’s modulus: 3.8 MPa for cork, 10.5 MPa for expanded PE, 5.38 MPa for Polyform and 1.54 MPa for Nora.

The results of measuring of hardness (Shore A) all six samples show that the highest values of hardness exhibit solid polymers, PE and PP (more than 93). They are followed by the cork with the hardness of 63.6. All three foamed polymers have much lower hardness: The hardness value for PEfoam is 32.4, for Polyform 28.9 and for Nora only 15.

### 3.5. Bacterial Adhesion Extent

**Figure 5:**
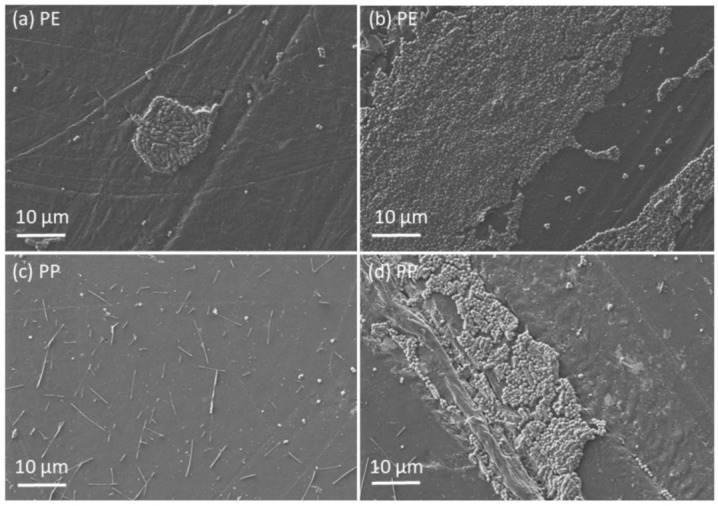
FE-SEM micrographs of the surface of solid polymer samples; bacterial adhesion on (**a**) PE–smooth surface, (**b**) PE–damaged surface, (**c**) PP-smooth surface and (**d**) PP–damaged surface.

**Figure 6:**
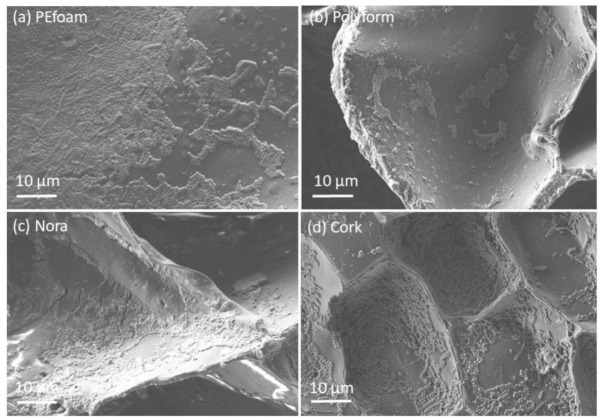
FE-SEM micrographs of the surface of foamed polymer samples; bacterial adhesion on (**a**) PEfoam, (**b**) Polyform, (**c**) Nora and (**d**) cork.

SEM microscopy was used to make micrographs of samples and evaluate the bacterial adhesion extent. In Figure 5 and Figure 6 micrographs of material surfaces with adhered bacteria are shown. The images were taken after 10 h of incubation. Quantitative analysis of micrographs was not performed because the bacteria preferentially adhered within the cavities of foamed materials, which made counting unreliable. Foamed materials (Figure 6) have a higher specific surface which offers more attachment points for the bacteria. Therefore, plain and solid materials (Figure 5) generally have lower bacteria coverage compared to the foamed ones. It was found that for solid polymer materials only in the surface defects and cracks the larger bacterial adhesion was detected (see Figure 5b,d)

### 3.6. Color Parameters

**Figure 7:**
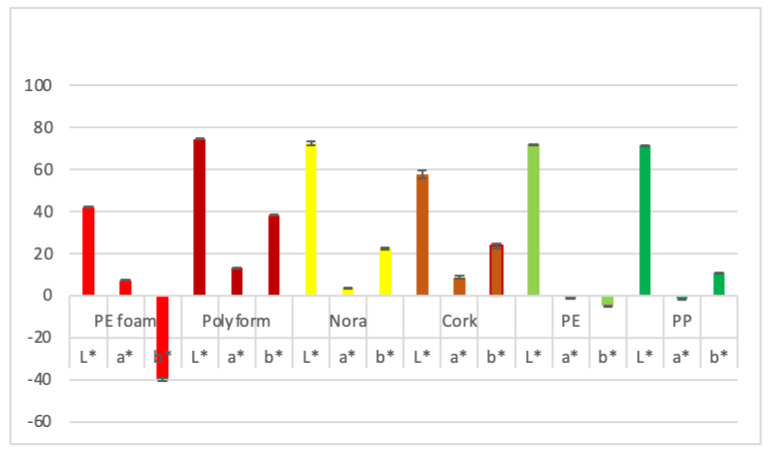
CIE color L*, a* b* parameters of material surfaces.

As seen from Figure 7 orthotic materials PEfoam, Polyform, Nora, cork, PE and PP. PE and PP exhibit similar L* values and consequently share similar lightness. Cork has lower L* value and PEfoam the lowest which ranks both as significantly darker. Polyform has the highest a* value, followed by cork and PEfoam. PE and PP exhibit slightly negative a* values that results in a more green color as compared to redish color of other samples. The highest b* value (yellow appearance) was recorded for cork, followed by Nora and PP, negative values (bluish appearance) were recorded for PE and PEfoam that had the lowest value at −39.8. In general, Polyform was among the lightest and the yellowish while PP was among the lightest and the greenish. On the other side, PEfoam was among the darkest and the bluish.

**Figure 8:**
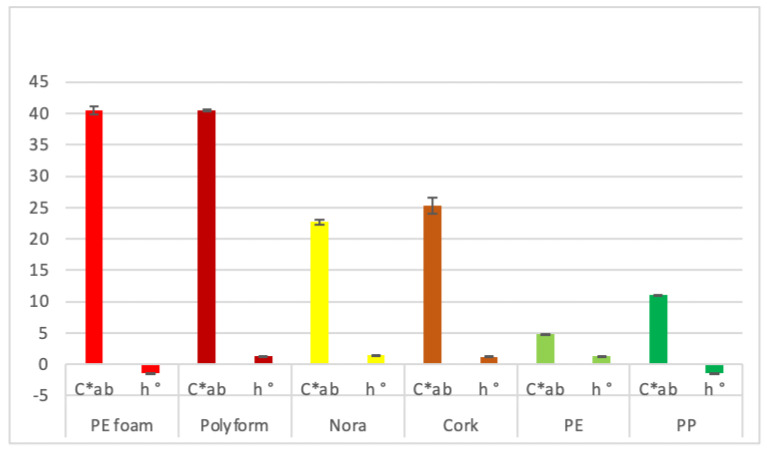
CIE color C*ab and h° parameters of material surfaces.

Polyform and PEfoam orthotic materials have the highest colorfulness as demonstrated by higher C*ab values (40.5), see Figure 8. Significantly lower colorfulness values were recorded for cork and Nora materials, 25.2 and 22.7 respectively. PP and PE have the lowest colorfulness 11.0 and 4.9 respectively. With regard to h° measurements, all values amount to around zero, a threshold that signifies red color. Slightly positive h° values ranged from 1.2 to 1.4 for cork, PE, Nora and Polyform orthotic material. PP and PEfoam have slightly negative values (−1.4). Besides being the lightest and the yellowish, Polyform was also among the most saturated in color, hence more colorful, as compared to other materials.

## 4. Discussion

As stated in the Introduction, the aim of this study was to extend investigations on orthotic and prosthetic surfaces like foamed polymeric materials: PEfoam, Polyform, Nora and cork, as well as solid materials: polyethylene and polypropylene, and examine their mechanical and antibacterial surface properties. We restrict our consideration to flat geometry only.

### 4.1. Surface Morphology/Topography and Roughness

Surface roughness is very important parameter for the adhesion of bacteria to material surfaces. It was found that very smooth surfaces do not favor bacterial adhesion, whereas very rough surfaces promote adhesion [25,26,27,28]. Irregularities like gaps and cracks present favorable places for bacteria to adhere because it protects them from external forces. The present study confirms the previous studies. Solid materials exhibit lover roughness than closed-cell materials. Consequently, the bacterial adhesion is larger on closed-cell surfaces (see Figure 5 and Figure 6).

### 4.2. Zeta Potential

*S. aureus* is negatively charged as with most bacteria [26,29]. Positively charged surfaces attract negatively charged bacteria whereas negatively charge surfaces repel negatively charged surfaces. The surfaces used in this study are negatively charged with the zeta potentials −53 mV, −65 mV and −69 mV for cork, PE and PP, respectively. For foamed materials, the zeta potential could not be measured. The zeta potential measurement indicates that the surfaces are highly negatively charge which contributes roughly the same repulsive force between bacteria and all surfaces considered.

### 4.3. Contact Angle Measurements

The surfaces with contact angles larger than 90° are called hydrophobic whereas the surfaces with contact angles smaller than 90° are named hydrophilic. We found that the hydrophobicity of the studied materials is diverse. The closed-cell materials are hydrophobic whereas solid materials are hydrophilic and hydrophobic. The results of hydrophobicity follow the rule that the increasing roughness makes the hydrophobic surface even more hydrophobic. Namely, PEfoam surface has the highest surface roughness and is the most hydrophobic material.

The hydrophobicity has an important consequence for initial bacterial adhesion and later bacterial colonization of the surfaces. We found the highest bacterial adhesion extent on hydrophobic closed-cell materials. The hydrophobic bacterial strains prefer to adhere to hydrophobic surfaces [26,30,31].

### 4.4. Mechanical Properties

The overview of mechanical properties shows huge differences between solid polymers and foamed polymeric materials. The Young’s modulus and the maximum tensile stress for solid PE and PP are much larger compared to the foamed materials, which is in line with the results in literature [1,12]. Young’s modulus and the maximum tensile stress of selected samples of solid polymers are 1380–2250 MPa and 28.3–33 MPa, respectively, while for foamed polymeric samples values vary between 1.54 MPa and 43.8 MPa (Young’s modulus) and between 0.898 MPa and 2.03 MPa (maximum tensile stress). The maximal strain at break was determined for the PE sample, which indicates a good material for long-lasting usage. The differences are found also in shapes of the stress/strain curves among PE, PP and foamed materials.

The highest and almost the same values of hardness exhibit solid polymers, PE and PP (Shore A = 93.4 and 93.2, respectively). All foamed samples have much lower hardness (Shore A from 15.0 to 63.6) which indicated that foamed samples are more comfortable to wear in the case of contact with skin.

Due to the strong effect of many structural and processing parameters of solid [4,5] as well foamed [6,8] polymers on mechanical properties, it is hard to compare the values of determined tensile properties of samples with those found in literature.

### 4.5. Bacterial Adhesion Extent

The bacterial adhesion was evaluated from a series of SEM micrographs (Figure 5 and Figure 6). The pronounced bacterial adhesion was observed on closed-cell materials whereas on solid materials less pronounced bacterial adhesion was observed. Generally, bacterial adhesion extent depends on the roughness, the zeta potential and the hydrophobicity as well as on bacteria surface properties [26,27,32]. In this study the driving force for the bacterial adhesion was the increasing roughness and increasing hydrophobicity. This study shows that solid materials minimize bacterial adhesion and thus improve the comfort of users. Studies of bacterial adhesion on particular materials have been conducted [33,34]. On the contrary, our study is a holistic approach in which we consider the most important materials used in orthotics and prosthetics.

Beside *S. aureus, S. epidermidis* is also part of the skin microbiota. Regarding adhesion properties both strains have similar adhesion properties and one can expect that both microorganisms qualitatively adhere in similar manner to the surfaces [35]. The main driving force for the adhesion of these strains to the material surfaces are their characteristics [36].

### 4.6. Color Parameters

As reported by Del Bino and Bernerd [37], L* value representing lightness of human skin ranges from 69 for very light color to 35 for dark color. Red/green intensity representing a* value ranges from 3.7 for very light to 10 for brown skin. Yellow/blue intensity representing b* value ranges from 14.5 for very light, to 20 for tan and 11.6 for dark skin [37]. Orhthosis color should be as close as possible to skin color in order to fulfil patient requirements. Results of our investigation show L*values of Polyform, Nora, polyethylene and polypropylene around 75, all matching very light skin. Cork orthotic material has L* value 57 matching tan skin, while PEfoam matches closely dark brown skin. Regarding a* value, PEfoam, Polyform and cork match closely intermediate to dark skin color and Nora matches very light skin color [37]. PE and PP have both slightly negative a*values representing green notes that are out of range for common skin color. CIE skin color parameter b* ranges according to skin type from 11 to 20 [37]. Our results show that Polyform with value of 40 is very intensive yellow and out of range for common skin types. Nora and cork match tan skin type, polypropylene matches dark skin. Polyethylene and PEfoam have bluish notes the later orthotic material is with value of −40 intensive blue and out of range for any skin type.

Chroma C*ab values and hue angle (h°) as calculated from results of [37] are presented in Table 4. Comparing C*ab values and hue angle (h°) to basic L*, a* and b* parameter, we notice quite narrow ranges for both parameters. C*ab ranges from 14 to 22 and h° from 0.92 to 1.32. PEfoam and Polyform have higher values (40) meaning more different from grey color. Nora and cork C*ab values resemble tan and brown skin while polyethylene and polypropylene have both lower values as common skin types. Hue angle values of Polyform, cork and polyethylene matches that of common skin [37]. Polypropylene and PEfoam have slightly negative values but nevertheless all hue angle values correspond to red color.

## 5. Conclusions

In this study, the impact of closed-cell structure of polymers on mechanical and antibacterial surface properties was investigated. The surface topography, roughness, hydrophobicity, and zeta potential were measured, and from the SEM micrographs the bacterial adhesion extent was estimated. We showed that the highest bacterial adhesion was on closed-cell polymer materials. The reason is the pronounced roughness and hydrophobicity of closed-cell polymers. The mechanical properties exhibit that foamed polymeric materials have lower Young’s modulus and lower maximum tensile strength compared to solid polymers samples. This study helps in understanding which orthotic or prosthetic materials reduce bacterial adhesion. Basic CIE L*, a* and b* values of orthotic and prosthetic materials differed from that of skin although calculated hue angle resemble vales for human skin.

We plan to modify polymeric properties in such a way that we can ensure the lowest bacterial adhesion with the optimal mechanical properties.

## Figures and Tables

**Table 1:** List of materials tested.

Designation	Material	Type	Product Name	Density (g/cm^3^)
PEfoam	Polyethylene	foamed	no data	0.125
Polyform	Polyethylene	foamed	Polyform	0.149
Nora	Poly(ethylene-vinyl acetate)	foamed	Nora	0.075
Cork	Cork-natural polymeric composite material	foamed	Cork	0.103
PE	Polyethylene	solid	Polyethylene	0.945
PP	Polypropylene	solid	Polypropylene	0.916

**Table 2:** Surface free energies.

Material	Surface Free Energy/mJm^−2^
PEfoam	16.8
Polyform	22.4
Nora	30.8
Cork	22.4
PE	33.8
PP	30.5

**Table 3:** The mean values with the standard deviations of Young’s modulus (E), the tensile stress at yield (σ_Y_), the tensile strain at yield (ε_Y_), the maximum tensile stress (σ_M_), the nominal strain at maximum tensile stress (ε_M_) and the strain at break (ε_B_), and hardness (Shore A) of 6 selected orthotic and prosthetic materials.

	E (MPa)	σ_Y_ (MPa)	ε_Y_ (%)	σ_M_ (MPa)	ε _M_ (%)	ε _B_ (%)	Hardness (Shore A)
PEfoam	10.5 ± 1.3			1.16 ± 0.02	86.0 ± 7	87.8 ± 6.7	32.4 ± 0.5
Polyform	5.38 ± 0.35			2.03 ± 0.04	156 ± 3	157 ± 3.2	28.9 ± 0.2
Nora	1.54 ± 0.06			1.27 ± 0.10	96.0 ± 6	-	15.0 ± 0
Cork	43.8 ± 11.8			0.898 ± 0.249	3.00 ± 1	-	63.6 ± 0.9
PE	1380 ± 24	21.0	10.0	28.3 ± 0.9	470 ± 10	471.7 ± 9.8	93.4 ± 0.5
PP	2250 ± 18	33.0 ± 0.3	4.00 ± 0	33.0 ± 0.3	4.00 ± 0	59.0 ± 26	93.2 ± 0.4

**Table 4:** Values for C*ab and h° for the six groups of skin color as calculated from data of [37].

	C*ab	h°
Very light	14.96	1.32
Light	18.76	1.19
Intermediate	20.11	1.19
Tan	22.58	1.11
Brown	22.58	1.11
Dark	14.56	0.92

## Data Availability

Not applicable.

## Supplementary Materials

The following are available online at https://www.mdpi.com/article/10.3390/ma14226877/s1, Figure S1. Typical stress-strain curves of paddle-shaped samples made of (**a**) PEfoam, (**b**) Polyform, (**c**) Nora, (**d**) cork, (**e**) PE and (**f**) PP.
