# Supplementary material for: Biophysical Properties of Foamed and Solid Polymers Used in Orthotics and Prosthetics
Source: Materials (Basel). 2021 Nov 15;14(22):6877. doi: 10.3390/ma14226877 (PMC8619838; doi:10.3390/ma14226877)
Supplement: Supplementary file 1 [file materials-14-06877-s001.zip › materials-1383767-supplementary.pdf]

Supplementary information

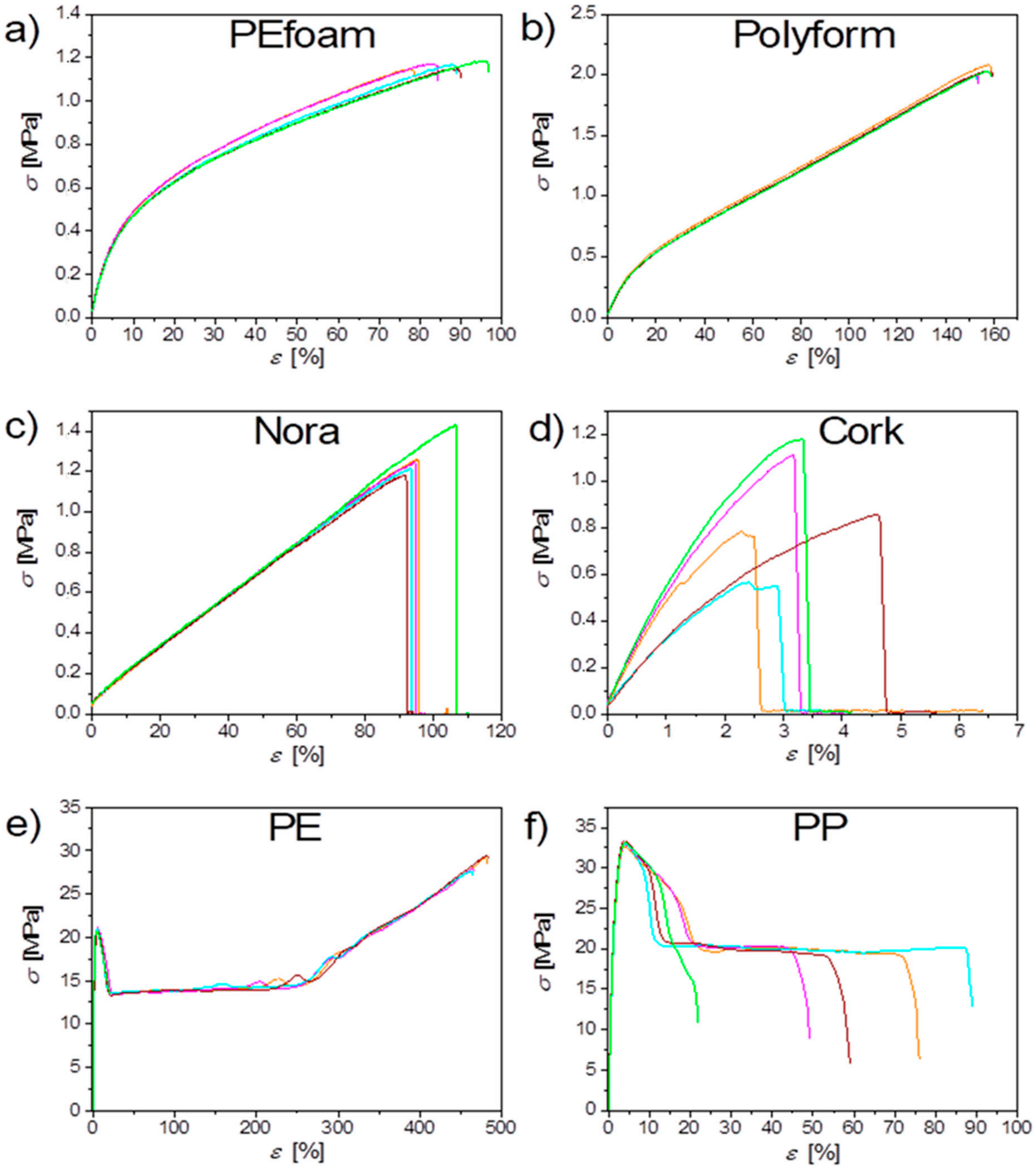

Figure S1. Typical stress-strain curves of paddle-shaped samples made of (**a**) PEfoam, (**b**) Polyform, (**c**) Nora, (**d**) Cork, (**e**) PE and (**f**) PP.
